# Microbial network and fermentation modulation of Napier grass and sugarcane top silage in southern Africa

**DOI:** 10.1128/spectrum.03032-23

**Published:** 2023-12-12

**Authors:** Zhumei Du, Seishi Yamasaki, Tetsuji Oya, Damiao Nguluve, Denise Euridse, Benedito Tinga, Felicidade Macome, Yimin Cai

**Affiliations:** 1 College of Animal Science and Technology, Yangzhou University, Yangzhou, China; 2 Japan International Research Center for Agricultural Sciences (JIRCAS), Tsukuba, Ibaraki, Japan; 3 Agricultural Research Institute of Mozambique, Matola, Mozambique; State Key Laboratory of Microbial Resources, Institute of Microbiology, Chinese Academy of Sciences, Beijing, China

**Keywords:** fermentation modulation, lactic acid bacteria screening, microbial co-occurrence network, PacBio SMRT, tropical silage

## Abstract

To effectively alleviate the shortage of animal feed in the tropics, we applied a screening lactic acid bacteria (LAB) strain (*Lactiplantibacillus plantarum* MO1) and a commercial inoculant (*L. plantarum* LP1) to prepare silage from early flowering Napier grass and after harvest sugarcane tops in southern Africa. The microbial community structure, co-occurrence network, and fermentation characteristics of silage were analyzed using PacBio single-molecule real-time sequencing technology. Strain MO1, isolated from corn silage, was characterized by strong acid tolerance and the ability to produce lactic acid. Before ensiling, the dominant microbial community in both Napier grass and sugarcane top were Gram-negative harmful microbes. After ensiling, the main microbial community shifted from Gram-negative to Gram-positive bacteria, and the microbial alpha diversity of all silages significantly decreased (*P* < 0.05). Compared to the control, fermentation of the LAB-treated silages was accelerated and *L. plantarum* became the dominant microbial community, while the lactic acid content increased (*P* < 0.05) and the pH was reduced (*P* < 0.05). In the MO1-treated silages, the relative abundance of LAB was markedly increased and formed a co-occurrence microbial network with *L. plantarum* as the dominant microorganism. It also led to the global and overview maps, as a special class of metabolic pathways in the Kyoto Encyclopedia of Genes and Genomes database was enhanced and the carbohydrate metabolism and amino acid metabolism pathways were weakened, thereby improving the flavor and quality of the silages compared to the LP1-treated ones. Strain MO1 can modulate the microbial community and metabolic pathways and achieve clean fermentation. It is of great significance to alleviate feed shortage and promote sustainable production of livestock.

**IMPORTANCE:**

Feed shortage in the tropics is a major constraint to the production of livestock products such as milk and meat. In order to effectively utilize of local feed resources, the selected lactic acid bacteria (LAB) strain was used to prepare Napier grass and sugarcane top silage. The results showed that the two silages inoculated with LAB formed a co-occurrence microbial network dominated by *Lactiplantibacillus* during the fermentation process, regulated the microbial community structure and metabolic pathways, and improved the silage fermentation quality. This is of great significance for alleviating feed shortage and promoting sustainable production of livestock.

## INTRODUCTION

Mixed agricultural production systems in Africa usually include food and livestock production activities. In these systems, small-scale farmers grow crops and harvest grains as staple foods for locals while also raising livestock (1). Crop by-products are the main source of roughage for ruminants, including cattle, sheep, and goat (2, 3). In Africa, there is a shortage of concentrate for livestock (4). The main feeding method of ruminants is grazing on natural grassland and using crop straws as supplementary feed (5). If low-quality feed cannot meet the nutritional requirements of livestock, there will be a decline in the production capacity of animal products, such as milk and meat, thereby affecting people’s living standards (6, 7). Tropical crops and grasses generally grow during the rainy season, and most crop by-products are produced during the harvest season before the beginning of the dry season. Some straws are used as feed for ruminants, but most of them are ploughed into the soil or burned (8). This not only results in a waste of biomass resources but also has a negative impact on the environment and global warming (9). Therefore, effective use of local feed resources is of strategic significance to promote animal production and prevent global warming (10). In this sense, the production of high-quality silage with a storage function is important to alleviate the shortage of feed in the dry season (11).

Silage is a stored feed prepared by lactic acid fermentation using high-moisture feed crops, grasses, or agricultural by-products (12). In the tropics, it can be produced in the rainy season and used in the dry season (13). The success or failure of silage fermentation depends on the type and fermentation capacity of lactic acid bacteria (LAB). During the silage process, LAB can convert water-soluble carbohydrate (WSC) into lactic acid, thereby lowering the pH, inhibiting the growth of harmful bacteria, and enabling long-term storage of forage (14). Therefore, screening excellent LAB strains is key for successful preparation of high-quality silage.

Napier grass (*Pennisetum purpureum* Schumach) belongs to the genus *Pennisetum* in the *Gramineae* family and is an important forage resource in the tropics (15). Generally, Napier grass has a high biomass; can adapt to various soil types, fertility levels, and weather conditions; and is widely cultivated in Africa for animal production, but there are fewer reports on silage fermentation and long-term storage (5). Sugarcane (*Saccharum officinarum* L.) is a perennial herb of the genus *Saccharum* in the *Gramineae* family (16). It is an important tropical crop for the sugar industry, with the main part of its stem used to process sugar juice (17). The sugarcane top occupies 15%–25% of the aboveground part of the plant and cannot be used for sugar production (18). It is usually exposed in the field as a crop residue (8). After drying, due to lignification, the palatability and digestibility to livestock are markedly reduced, resulting in most of the residue being treated by burning. In the tropics, sugarcane by-product resources are abundant and cheap. The fresh sugarcane top after harvest can be used to make low-cost silage, thereby helping to solve the problem of the shortage of feed resources in the dry season.

Generally, silage fermentation is a dynamic process characterized by microbial community succession and metabolite changes (19). The traditional microbial culture method can only isolate and identify certain cultivable microorganisms during the silage process and does not fully reflect the microbial community structure that affects the silage fermentation mechanism (20). In recent years, Pacific Biosciences (PacBio) single-molecule real-time (SMRT) third-generation sequencing technology has been widely used in fermented food and feed (21 – 24); this method can provide more detailed information about the structure and diversity of microbial communities at the species level. However, there is little information available regarding the screening of LAB or the use of third-generation sequencing to analyze the mechanism of silage fermentation in Africa. In this study, we screened natural LAB isolated from forages in Mozambique and prepared silage using local feed resources. To reveal the fermentation mechanism of silage, SMRT sequencing technology was used to study the microbial community, co-occurrence network, and fermentation characteristics.

## RESULTS

The pH, lactic acid buffer capacity (LBC), microbial population, chemical and protein composition, energy, and macro-mineral values of Napier grass and sugarcane top before ensiling are shown in Table 1. The dry matter (DM) content of the Napier grass and sugarcane top ranged from 26% to 29%. The organic matter (OM), ether extract (EE), acid detergent fiber (ADF), and acid detergent lignin (ADL) contents of both materials did not show marked differences. The LBC, crude protein (CP), neutral detergent fiber (NDF), WSC, true protein (TP), net energy for maintenance (NEm), net energy for lactation (NEl), and net energy for gain (NEg) were higher (*P* < 0.01), but the pH, non-protein nitrogen (NPN), and macro-mineral including calcium, phosphorous, magnesium, potassium contents were low (*P* < 0.01) in sugarcane top than those in Napier grass. The microbial counts were 10^6^ colony-forming units per gram (cfu/g) of fresh matter (FM) for aerobic bacteria, 10^3^–10^5^ for coliform bacteria, 10^3^–10^4^ for LAB, 0–10^3^ for yeast, and below the detectable level (<10^2^) for clostridia and molds in both forages.

**TABLE 1:** pH, LBC, chemical and protein composition, energy component, macro-mineral, and microbial population of material^*a*^ ^,^ ^*b*^

Items	Napier grass	Sugarcane top	SEM	*P*-value
pH	7.01^a^	6.53^b^	0.02	<0.001
LBC (mEq/kg of DM)	528.08^b^	1,772.40^a^	7.50	<0.001
Chemical composition				
DM (%)	28.39	26.68	1.34	0.42
OM (% of DM)	88.18	88.35	0.34	0.74
CP (% of DM)	5.28^b^	6.66^a^	0.18	0.006
EE (% of DM)	1.29	1.68	0.13	0.10
NDF (% of DM)	61.04^b^	71.04^a^	1.01	0.002
ADF (% of DM)	36.85	39.63	0.74	0.06
ADL (% of DM)	5.31	4.77	0.23	0.17
WSC (% of DM)	4.22^b^	7.85^a^	0.10	<0.001
Protein composition (% of DM)				
NPN	1.69^a^	2.10^b^	0.16	0.01
TP	3.68^b^	3.46^a^	0.05	0.02
Energy component (Mcal/kg of DM)				
NEm	0.64^b^	0.95^a^	0.02	<0.001
NEl	0.61^b^	0.92^a^	0.03	<0.001
NEg	0.35^b^	0.41^a^	0.02	0.02
Macro-mineral (g/kg of DM)				
Calcium	0.41^a^	0.25^b^	0.02	0.004
Phosphorous	0.41^a^	0.14^b^	0.01	<0.001
Magnesium	0.50^a^	0.17^b^	0.02	<0.001
Potassium	2.06^a^	1.70^b^	0.05	0.009
Microbial population (Log _10_ cfu/g of FM)				
Lactic acid bacteria	3.76^b^	4.49^a^	0.05	0.008
Aerobic bacteria	6.41	6.04	0.44	0.77
Coliform bacteria	5.30^a^	3.53^b^	0.18	<0.001
Clostridia	ND	ND	ND	ND
Yeast	ND	3.23	0.25	0.69
Mold	ND	ND	ND	ND

^*a*^ Data are means of three samples; means in the same row followed by different lowercase letters (a and b) differ (*P* < 0.05).

^*b*^ SEM, standard error of the mean; LBC, lactic acid buffering capacity; DM, dry matter; OM, organic matter; CP, crude protein; EE, ether extract; NDF, neutral detergent fiber; ADF, acid detergent fiber; ADL, acid detergent lignin; WSC, water-soluble carbohydrate; NPN, non-protein nitrogen; TP, true protein; NEm, net energy for maintenance; NEl, net energy for lactation; NEg, net energy for gain; cfu, colony-forming unit; FM, fresh matter; ND, not detected.

The characteristics of LAB isolated from silage and inoculant are shown in Table 2. Strains MO1, MO4, and LP1 were Gram-positive and catalase-negative bacteria that produce the more lactic acid from glucose. Strains MO1 and LP1 were homo-fermentative LAB and produced lactic acid as DL-isomer, while strain MO4 was hetero-fermentative LAB and produced lactic acid as D-isomer. Strain MO1 can grow well at 45°C and pH 3.0, but the strains LP1 and MO4 did not. The production ability of lactic acid in lactobacilli de Man, Rogosa, and Sharpe (MRS) broth were in order of strains MO1, LP1, and MO4. The similarity of 16S rDNA between isolate and their type strains was more than 99.88%, strains MO1 and LP1 can be identified as *Lactiplantibacillus plantarum*, and strain MO4 belonged to *Weissella confusa*.

**TABLE 2:** The characteristics of lactic acid bacteria isolated from silage and inoculant^*a*^

Characterization	*Lactiplantibacillus plantarum* MO1	*Weissella confusa* MO4	*Lactiplantibacillus plantarum* LP1
Source	Corn	Napier grass	Inoculant
Gram stain	+	+	+
Catalase reaction	−	−	−
Cell form	Rod	Cocci	Rod
Spore	−	−	−
Fermentation type	Homo	Hetero	Homo
Optical isomers of lactic acid	DL	D(-)	DL
Growth at temperature			
40°C	+	+	+
45°C	+	−	W
50°C	−	−	−
Growth at pH			
3.0	+	−	W
3.5	+	−	+
4.0	+	−	+
4.5	+	W	+
5.0	+	+	+
Lactic acid production in MRS broth (OD 620)	2.2	0.86	2.0
Similarity of 16S rDNA between isolate and type strain (%)	99.88	99.95	100

^*a*^ +, positive reaction; −, negative reaction; W, weak positive reaction; OD, optical density.

The fermentation quality and microbial population of Napier grass and sugarcane top silages are shown in Table 3. After 60 days of ensiling, the DM contents of the Napier grass and sugarcane top silages were similar, ranging from 27% to 29%, which was not affected (*P* = 0.06 or 0.07) by forage (F), additive (A), and forage × additive (F × A). Compared to the control silages, all of the MO1- and LP1-treated of Napier grass and sugarcane top silages were well preserved, with lower (*P* < 0.05) pH, acetic acid, and ammonia nitrogen (NH_3_-N) content and higher (*P* < 0.05) lactic acid content. The strain MO1 had better fermentation quality with higher (*P* < 0.001) lactic acid and lower (*P* < 0.001) pH and NH_3_-N contents than inoculant LP1 in both silages. The propionic acid and butyric acid contents in LP1 treatments were significantly lower (*P* < 0.001) than control, and these organic acids were below the detectable level in MO1 treatment in Napier grass and sugarcane top silages. The F influenced (*P* < 0.05) pH, acetic acid, propionic acid, butyric acid, and NH_3_-N of silage; A influenced (*P* < 0.05) all the fermentation quality indexes; and F × A influenced (*P* < 0.05) lactic acid, propionic acid, and NH_3_-N. In the Napier grass and sugarcane top silages, the LAB were dominant species as 10^6^–10^8^ cfu/g of FM in MO1 and LP1 treatments, while the aerobic bacteria were the main bacteria as 10^5^ in control. Compared to the control, the strains MO1 and LP1 in both silages significantly (*P* < 0.001) reduced the mold counts. The LAB counts were higher (*P* < 0.001) and aerobic bacteria counts were lower (*P* < 0.001) in MO1-treated silages than those in LP1-treated silages. The coliform bacteria and clostridia were below the detectable levels in MO1-treated of both silages. The F and F × A influenced (*P* < 0.01) LAB, aerobic bacteria, coliform bacteria, and clostridia, except for yeast and mold; A influenced (*P* < 0.001) all the microbial population indexes.

**TABLE 3:** The fermentation quality and microbial population of Napier grass and sugarcane top silage^*a*^ ^,^ ^*b*^

Item	Napier grass silage			Sugarcane top silage			SEM	*P*-value		
	Control	MO1	LP1	Control	MO1	LP1		F	A	F × A
Fermentation quality										
DM (%)	27.03	26.85	28.76	28.95	27.70	28.03	0.11	0.06	0.07	0.06
pH	5.32^a^	3.92	4.43^b^	4.83^a^	3.80	4.15^b^	0.08	<0.001	<0.001	0.099
Lactic acid (% of FM)	0.64	1.26^a^	0.97^b^	0.55	1.43^a^	1.12^b^	0.05	0.07	<0.001	0.03
Acetic acid (% of FM)	0.52^a^	0.22	0.34^b^	0.45^a^	0.21	0.26^b^	0.03	0.03	<0.001	0.39
Propionic acid (% of FM)	0.15^a^	ND	0.08^b^	0.08^a^	ND	0.06^b^	0.01	<0.001	<0.001	0.006
Butyric acid (% of FM)	0.33^a^	ND	0.21^b^	0.26^a^	ND	0.17^b^	0.01	0.006	<0.001	0.07
NH_3_-N (g/kg of FM)	0.45^a^	0.16	0.38^b^	0.58^a^	0.24	0.36^b^	0.02	<0.001	<0.001	0.001
Microbial population (log _10_ cfu/g of FM)										
Lactic acid bacteria	3.80	8.23^a^	6.82^b^	3.80	8.20^a^	7.50^b^	0.08	0.004	<0.001	<0.001
Aerobic bacteria	5.83^a^	3.24	4.53^b^	5.83^a^	3.05^b^	3.23^b^	0.07	<0.001	<0.001	<0.001
Coliform bacteria	4.82^a^	ND	3.37^b^	4.80^a^	ND	2.95^b^	0.04	<0.001	<0.001	<0.001
Clostridia	4.34^a^	ND	3.40^b^	4.33^a^	ND	3.12^b^	0.03	<0.001	<0.001	<0.001
Yeast	ND	3.04^b^	3.51^a^	ND	3.12^b^	3.57^a^	0.04	0.14	<0.001	0.53
Mold	3.26^a^	ND	ND	3.28^a^	ND	ND	0.02	0.60	<0.001	0.75

^*a*^ Data are means of three samples; means in the same row followed by different lowercase letters (a–c) differ (*P* < 0.05).

^*b*^ MO1, *L. plantarum* MO1; LP1, *L. plantarum* LP1; SEM, standard error of the mean; F, forage; A, additive; F × A, the interaction of forage and additive; DM, dry matter; FM, fresh matter; NH_3_-N, ammonia nitrogen; cfu, colony-forming unit; ND, not detected.

General information of sequence and alpha diversity indexes of Napier grass and sugarcane top before and after ensiling is shown in Table 4. After optimization, the number of circular consensus sequence (CCS) in all treatments ranged from 10,327 to 12,022, with an average of 11,587. The alpha diversity indexes of Napier grass and sugarcane top were no great difference; after ensiling, their abundance-based coverage estimator (ACE), Chao1, Simpson, Shannon, and observed species indexes decreased (*P* < 0.05). For the alpha diversity indexes, the LAB-treated silages were lower (*P* < 0.05) than those of control silages, but the LP1-treated silages were higher (*P* < 0.05) than MO1-treated silages. The sequencing coverage of both materials and silages was greater than 0.99.

**TABLE 4:** General information of sequence and alpha diversity indexes of Napier grass and sugarcane top before and after ensiling^*a*^

Items	Napier grass	Sugarcane top	Napier grass silage			Sugarcane top silage		
			Control	MO1	LP1	Control	MO1	LP1
Number of CCS	11,564	11,954	12,022	11,769	10,327	11,575	11,977	11,510
Alpha diversity index								
ACE	418	426	370	339	360	359	305	341
Chao1	416	441	381	238	366	359	298	324
Simpson	0.97	0.95	0.95	0.85	0.94	0.97	0.87	0.92
Shannon	6.24	6.01	5.54	3.84	5.27	6.35	4.24	4.63
Coverage	0.99	0.99	1.00	0.99	0.99	1.00	0.99	1.00
Observed species	284	291	260	238	229	269	215	218

^*a*^ Data are means of the three replicate samples. MO1, *L. plantarum* MO1; LP1, *L. plantarum* LP1; CCS, circular consensus sequences; ACE, abundance-based coverage estimator.

**Fig 1:**
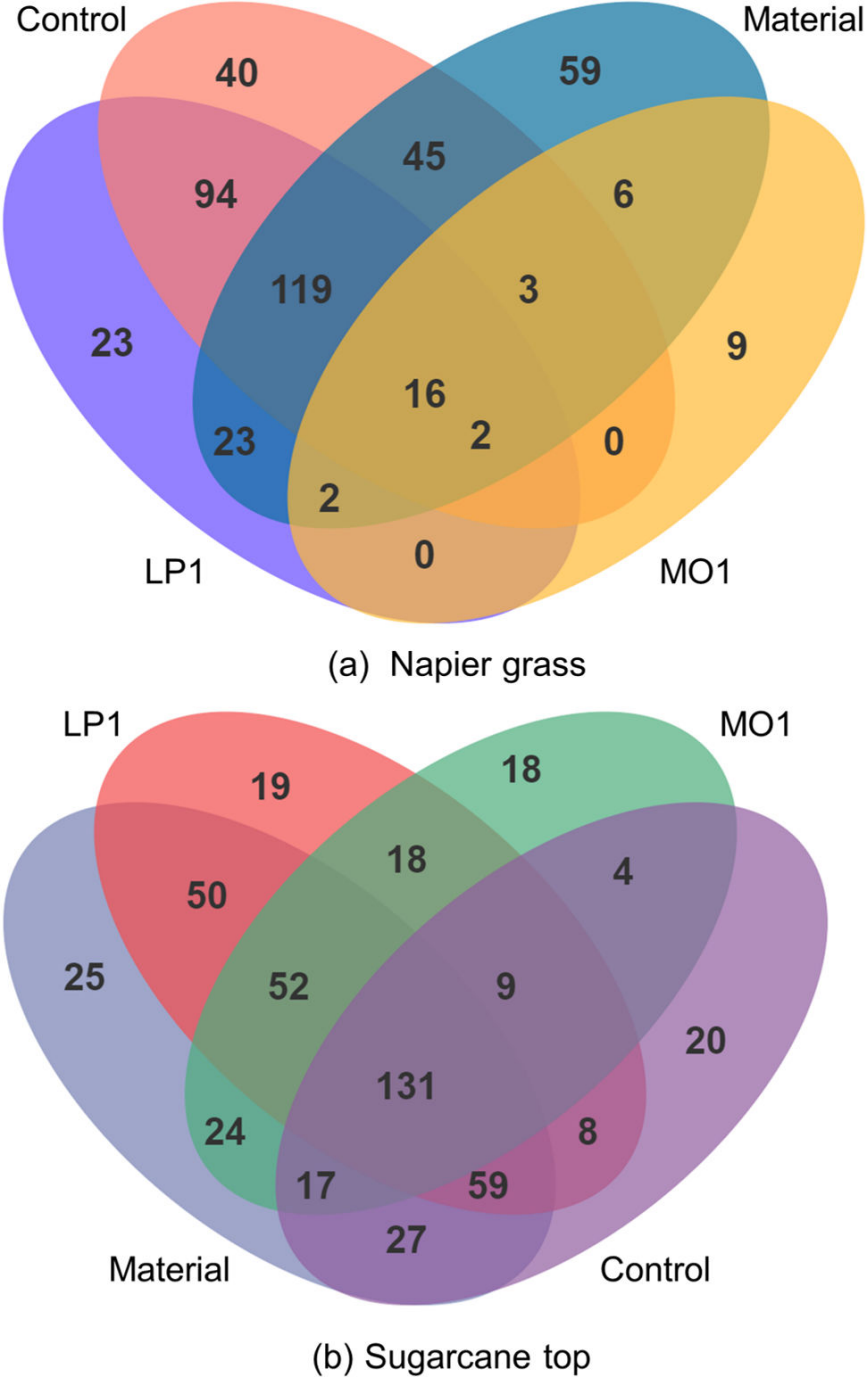
Venn diagram of OTU numbers at 97% sequence identity in Napier grass (**a**) and sugarcane top (**b**) before and after ensiling. OTU, operational taxonomic unit; MO1, *L. plantarum* MO1; LP1, *L. plantarum* LP1.

A Venn diagram of the operational taxonomic unit (OTU) numbers at 97% sequence identity in Napier grass and sugarcane top before and after ensiling is shown in Fig. 1. The dominant microbiome of Napier grass and silages (Fig. 1a) contained 16 shared OTUs as well as 9 and 59 unique OTUs, respectively. The dominant microbiome of sugarcane top and silages (Fig. 1b) contained 131 shared OTUs as well as 18 and 25 unique OTUs, respectively. The unique OTU in all silages was much lower than that in the materials before ensiling, especially the OTU in MO1 treatments which showed the lowest number among the two silages.

**Fig 2:**
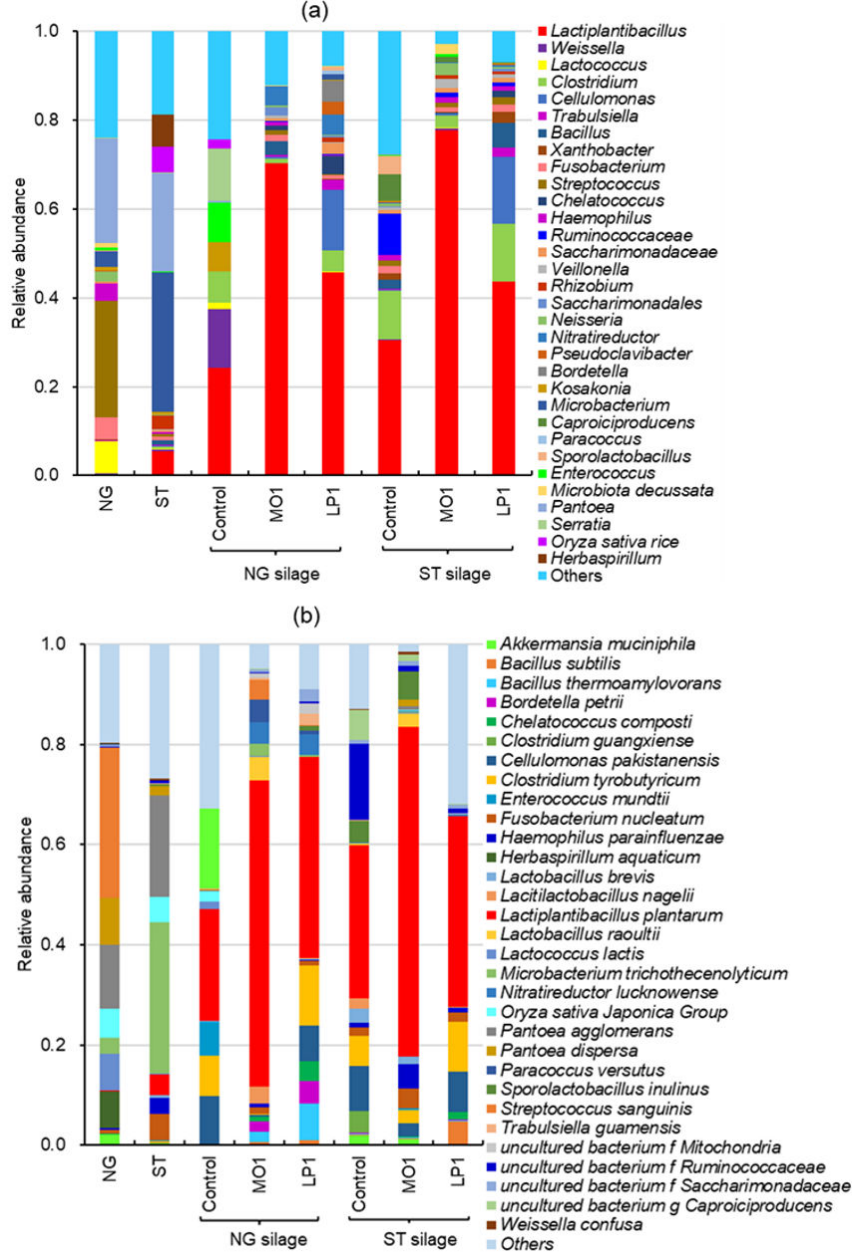
The relative abundance of the bacterial community at the genus (**a**) and species (**b**) levels in the NG and ST before and after ensiling. NG, Napier grass; ST, sugarcane top; MO1, *L. plantarum* MO1; LP1, *L. plantarum* LP1.

The relative abundances of the bacterial communities at the genus (a) and species (b) levels in the Napier grass and sugarcane top before and after ensiling are shown in Fig. 2. Before ensiling, the dominant genera were *Streptococcus* and *Pantoea* for Napier grass and *Microbacterium* and *Pantoea* for sugarcane top. The dominant species were *Streptococcus sanguinis* and *Pantoea agglomerans* in Napier grass and *Microbacterium trichothecenolyticum* and *P. agglomerans* in sugarcane top. After ensiling, *L. plantarum* dominated in all the Napier grass and sugarcane top silages and was observed at the highest abundance in the MO1-treated both silages. In the control of both silages, the second abundant genera were *Weissella* and *Ruminococcaceae*, including species of *W. confusa* and *Ruminococcaceae* spp. Also, there are a number of *Clostridium* (*Clostridium guangxiense*) in the control silages.

**Fig 3:**
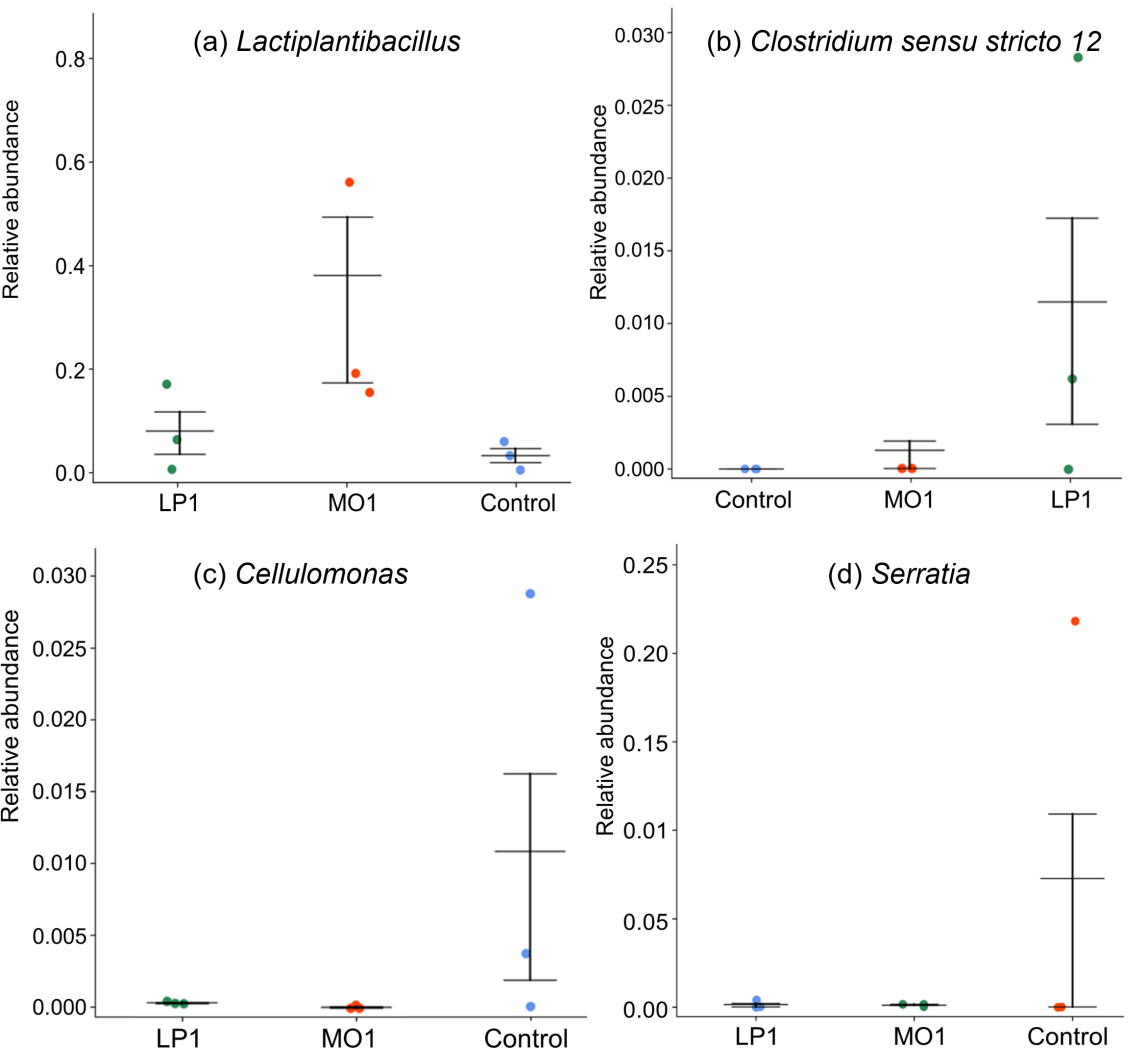
ANOVA four bacterial genera (a) *Lactiplantibacillus*, (b) *Clostridium sensu stricto* 12, (c) *Cellulomonas* and (d) *Serratia* with significant changes in Napier grass silage. LP1, *L. plantarum* LP1; MO1, *L. plantarum* MO1.

Analysis of variance (ANOVA) of four bacterial genera with significant changes in Napier grass silage is shown in Fig. 3. The relative abundance of *Lactiplantibacillus* was higher (*P* < 0.01), and that of *Clostridium sensu stricto 12*, *Cellulomonas*, and *Serratia* were lower (*P* < 0.01) in MO1- and LP1-treated silages than those in the control silage. The highest relative abundance of *Lactiplantibacillus* and the lowest that of *Clostridium sensu stricto 12*, *Cellulomonas*, and *Serratia* were found in MO1-treated silages.

**Fig 4:**
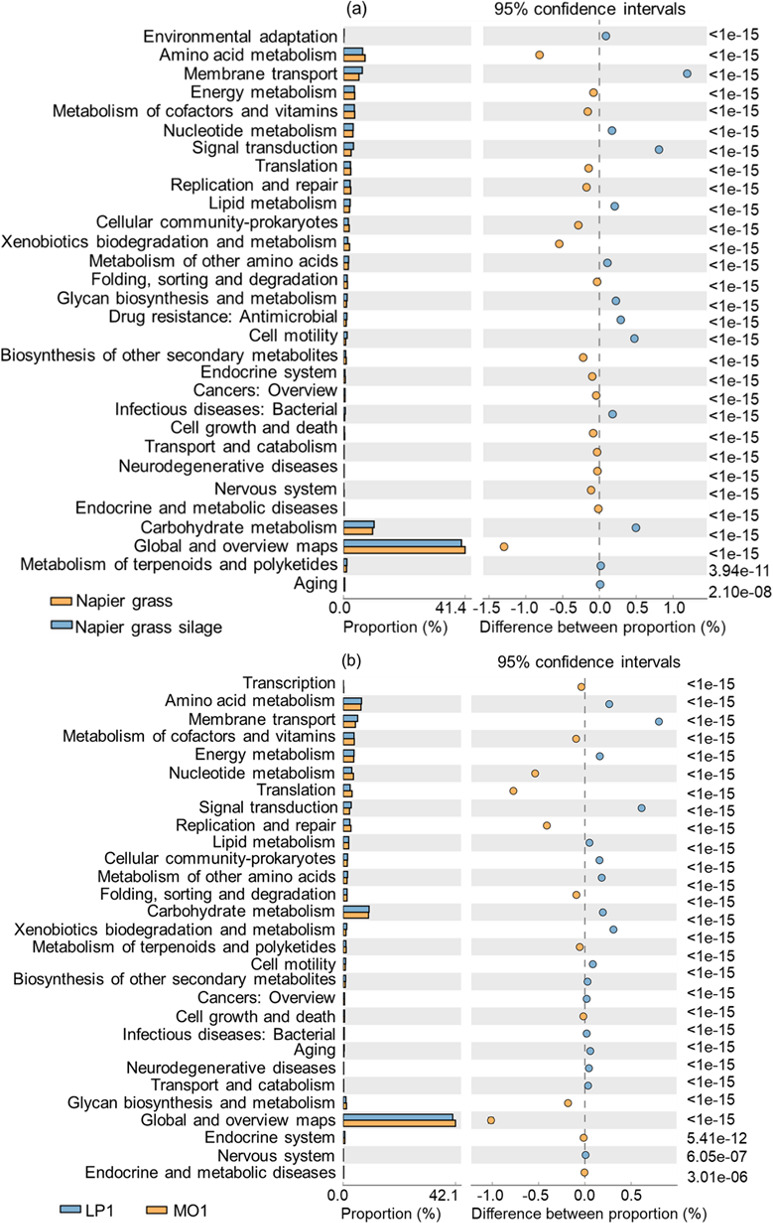
The impacted KEGG metabolic pathways in the Napier grass before and after ensiling (**a**) and LP1- and MO1-treated silage (**b**). KEGG, Kyoto Encyclopedia of Genes and Genomes; LP1, *L. plantarum* LP1; MO1, *L. plantarum* MO1.

The impacted Kyoto Encyclopedia of Genes and Genomes (KEGG) metabolic pathways in the Napier grass before and after ensiling (a) and LP1- and MO1-treated silage (b) are shown in Fig. 4. The global and overview maps (GOM), carbohydrate metabolism, and amino acid metabolism were the predominant metabolic categories in Napier grass and their silages. The metabolism pathway of GOM shared as the largest proportion in Napier grass and silages. The proportion of the carbohydrate metabolism pathway was higher (*P* < 0.01), whereas their amino acid and GOM metabolism pathways were lower (*P* < 0.01) in Napier grass silage than those in material (Fig. 4a). The proportion of the GOM metabolism pathway was higher (*P* < 0.01), whereas their carbohydrate and amino acid metabolism pathways were lower (*P* < 0.01) in MO1 treatment than those in LP1 treatment of Napier grass silage (Fig. 4b).

**Fig 5:**
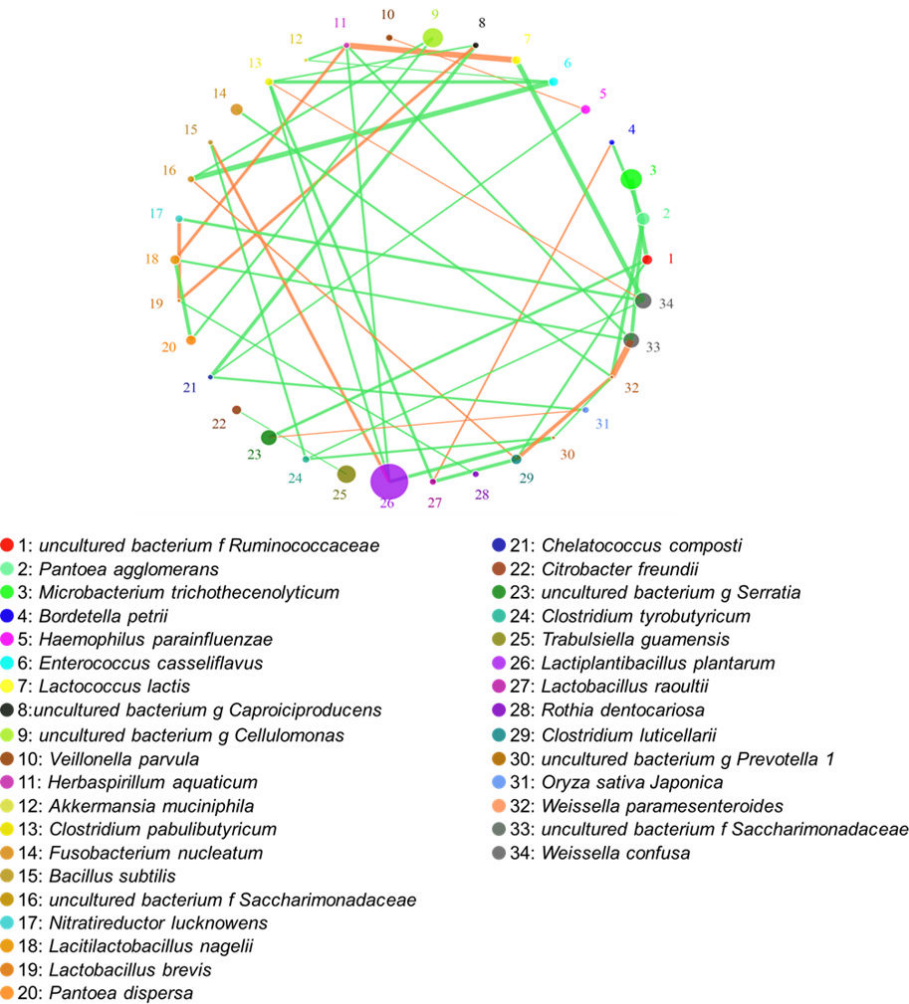
Microbial co-occurrence network at the species level related to silage fermentation of Napier grass. f, family; g, genus.

Microbial correlation network at the species level related to silage fermentation of Napier grass is shown in Fig. 5. The most abundant species in the Napier grass silage was *L. plantarum*, which was positively correlated with *Bacillus subtilis* and negatively correlated with *Herbaspirillum aquaticum* and *Clostridium pabulibutyricum*. The second abundant species was *M. trichothecenolyticum*, which was negatively correlated with the uncultured bacterium family *Ruminococcaceae* and *P. agglomerans*.

**Fig 6:**
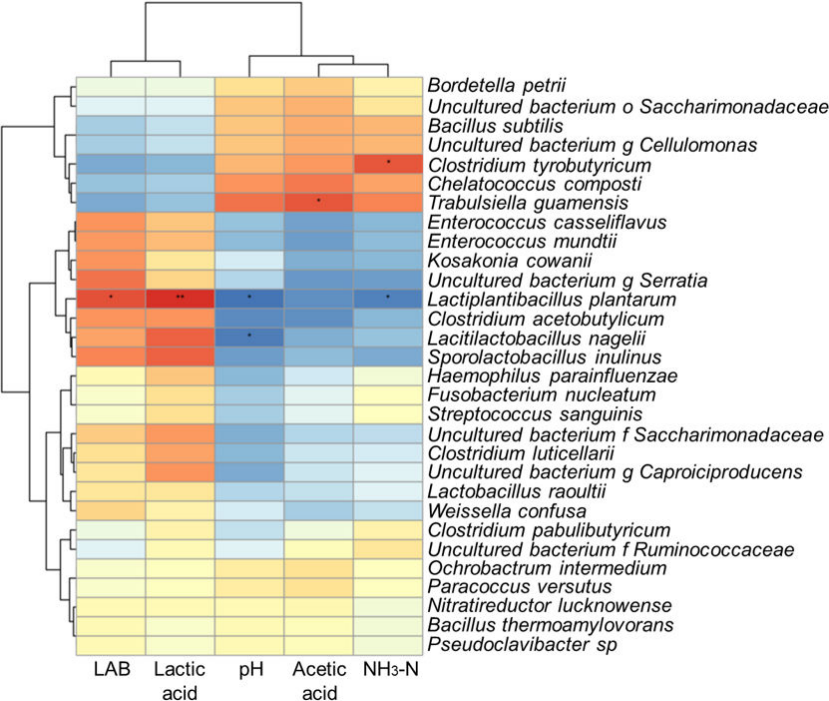
Correlation heatmap and hierarchical cluster analysis between bacterial community and terminal fermentation products in the Napier grass silage. LAB, lactic acid bacteria; NH_3_-N, ammonia nitrogen.

Correlation heatmap and hierarchical cluster analysis between bacterial community and terminal fermentation products in the Napier grass silage are shown in Fig. 6. Lactic acid and LAB were strongly and positively correlated with *L. plantarum*. The pH was negatively correlated with *L. plantarum* and *Lacitilactobacillus nagelii*. Acetic acid was positively correlated with *Trabulsiella guamensis*. The NH_3_-N was positively correlated with *Clostridium tyrobutyricum* and negatively correlated with *L. plantarum*.

## DISCUSSION

A shortage of feed in the dry season is the main factor restricting the production of tropical ruminants. Making the best use of existing local feed resources and reducing feed costs will play an important role in protecting the future interests of farmers and promoting sustainable agricultural development (25). Napier grass and sugarcane are the main grass and sugar crops cultivated in Africa, respectively (8, 26, 27). Except for the small amounts used as feed for ruminants, most of these by-products are incinerated before the next production season. In this study, fresh sugarcane tops after harvest contained certain nutrients and macro-minerals, and the CP, EE, NDF, NEm, NEl, and NEg contents were all higher than in Napier grass. This indicates that sugarcane tops, like Napier grass, can be used as high-quality roughage for tropical ruminants.

Generally, to prepare high-quality silage, at least 10^5^ cfu/g FM of LAB is required (14). However, the numbers of epiphytic LAB in forage crops and grasses are generally lower than 10^4^ cfu/g of FM. *Lactiplantibacillus* species that play an important role in the silage fermentation process, such as *L. plantarum* and *Lacticaseibacillus casei*, are generally present in amounts below the detection level. As shown in Table 1, the epiphytic LAB counts in the two forages were lower than 10^4^ cfu/g of FM, but the aerobic bacteria not conducive to silage fermentation were present in amounts higher than 10^6^ cfu/g of FM, indicating the need to add LAB to control the microbial community. Therefore, screening and using excellent LAB strains are key for successful preparation of silage.

LAB are Gram-positive and Gram-negative bacteria that produce lactic acid from glucose. They can be divided into rod and cocci shapes according to the cell morphology, and homo- and hetero-fermentation bacteria, according to the fermentation type (28). Lactic acid-producing cocci usually prefer a microaerobic and near-neutral pH environment. In the initial stage of silage fermentation, cocci can proliferate and produce a certain amount of lactic acid, which can create an acidic and anaerobic environment for the subsequent growth of lactobacilli (29). However, the cocci-shaped bacteria have a relatively low acid resistance, and with the progress of silage fermentation and decrease in pH, the population will decrease; most will eventually die (30, 31). In contrast, lactobacilli have high acid resistance and lactic acid production ability. They can proliferate vigorously for a long time in silage, produce a large amount of lactic acid, and improve the quality of fermentation (14). The strain MO1 used in this study is a homofermentative lactobacilli that can grow well at pH 3.0. Its lactic acid production capacity and high temperature tolerance are better than those of the inoculant strain LP1. MO4 is a representative isolated strain. It is a hetero-fermentative *Weissella* that cannot grow below pH 4.0. Its lactic acid production capacity is lower than that of MO1 and LP1 (Table 2). Through a 16S rRNA sequence analysis, MO1 was identified as *L. plantarum*. The physiological characteristics of this strain enable it to be applied in the high-temperature silage fermentation environment in the tropics, and it is expected to be developed as a microbial inoculant for silage production.

The microbial inoculant and screening strains used in this study were homo-fermentative LAB. They can grow well under low pH conditions, proliferate vigorously in the silage environment, and produce large amounts of lactic acid, thereby effectively inhibiting the growth of undesirable microorganisms during the silage process. Compared to LP1, strain MO1 has a lower pH tolerance and stronger lactic acid production capacity and can, therefore, improve the fermentation quality of silage. On the other hand, the poor quality of the control silage was due to the fermentation resulting from the epiphytic LAB and to a failure to lower the pH of the silage below 4.2, which resulted in the proliferation of butyric acid bacteria and other bacteria. These harmful microorganisms decomposed protein and reduced the fermentation quality of the silage. As shown in Table 3, the relatively high contents of butyric acid and NH_3_-N in the control silage supported this argument from the perspective of the fermentation products. As shown in Fig. 2, the low relative abundance of epiphytic LAB, which has strong fermenting ability in the material, and the high relative abundance of lactobacilli in the LAB-treated silage strongly supported this argument from the perspective of the microbial community.

Alpha diversity usually reflects the microbial abundance and species diversity of a sample. The Chao1 and ACE indexes are usually used to measure species abundance, while the Shannon and Simpson indexes measure species diversity (32). As shown in Table 4, fresh Napier grass and sugarcane tops had a high moisture content and contained an abundance of nutrients. The environmental microbes were suitable for crop growth, resulting in high alpha diversity and a large number of observed species. After ensiling, the microbial community shifted from Gram-negative to Gram-positive bacteria, and the LAB were finally transformed into the dominant community in silage fermentation. As a result, the ACE, Chao1, Shannon, and Simpson alpha diversity indexes and the number of species observed dropped sharply in all silages. Due to the addition of LAB, the succession process of microorganisms was accelerated, the lactic acid fermentation of silage was promoted, and the growth of other microorganisms was inhibited. This was also the main factor underlying the lower microbial abundance and diversity of MO1 than LP1 during the silage process.

Venn diagrams can show the number of common and unique OTUs between samples (19). Using OTUs for species classification, it is possible to analyze the common microorganisms in the individuals of each sample under different environmental conditions, including the silage fermentation process (20). As shown in Fig. 1, the conditions in the fresh materials before ensiling were good for microbial growth, leading to a high number of OTUs in Napier grass and sugarcane tops; this reflected the rich diversity of epiphytic microorganisms. After ensiling, due to the dual stress of the acidic and anaerobic environment formed by silage fermentation, aerobic Gram-negative bacteria with thin cell walls died quickly. Only a few acid-tolerant microorganisms, such as LAB and spore bacteria, were able to survive, resulting in a decline in the microbial community, including in terms of diversity. This was also the main reason why the microbial diversity was reduced in high-quality silage.

Silage fermentation is a dynamic process occurring during succession of the microbial community and changes in metabolites (33). The microbial community structure affects metabolic pathways, and metabolites restrict the growth of microorganisms. They interact to affect the fermentation quality of silage (3, 34). *S. sanguinis* and *P. agglomerans* were the main species in the Napier grass material (Fig. 2). *S. sanguinis* is a Gram-positive facultative anaerobic coccid bacterium (35). It is often present in the human oral cavity and is associated with the formation of dental plaque. It is rarely isolated in the silage environment. *P. agglomerans* is a ubiquitous Gram-negative bacteria usually isolated from the surface of plants and animal or human feces (36). The physiological and biochemical properties of the two bacteria and their roles in silage fermentation are still unclear; further study is needed. The dominant strain in sugarcane tops is *Microbacterium* spp., a Gram-positive bacteria that can grow in aerobic or weakly anaerobic environments, does not produce spores, is not acid tolerant, and can grow stably in the pH range of 5.0–10.0 (37). Because the pH of the sugarcane tops is almost neutral, it is suitable for the growth of this bacteria. In contrast, the abundance of LAB in both materials is low, but there are many harmful bacteria, indicating that Napier grass and sugarcane tops need to be mixed with excellent LAB strains. Measures can be taken to control the microbial community to improve silage fermentation.

In the control silage, some bacteria were not conducive to silage fermentation, such as *Rumenococcus* spp., *Cellulomonas pakistanensis*, and *C. guangxiense*. These bacteria may negatively affect the quality of silage fermentation (38). However, in both LAB-treated silages, *L. plantarum* was the dominant species, and the process of microbial succession was rapidly completed in the silage. A reasonable explanation for this phenomenon is that, during the fermentation process, *L. plantarum* can quickly respond to the dual stress of anaerobic and acidic environments to carry out lactic acid fermentation, thereby improving the quality of silage fermentation.

Various microorganisms are associated with silage fermentation, and they all affect the fermentation quality (8, 39). During the ensiling process, LAB can transform WSC into lactic acid, reduce the pH, inhibit the proliferation of harmful microorganisms, and promote the growth of LAB, thereby improving the fermentation quality of silage. The abundance of *Clostridium*, *Cellulomonas*, and *Serratia* in the control silage resulted in competition with LAB for nutrition, which was not conducive to silage fermentation. *Clostridium* are anaerobic bacteria with spores that can grow in an anaerobic environment, resulting in poor silage fermentation. The MO1-treated silage effectively inhibited the growth of these harmful microorganisms and promoted silage fermentation.

In the KEGG metabolic pathway enrichment analysis (Fig. 4), Napier grass and silage were the most important metabolic pathways according to GOM, followed by the carbohydrate and amino acid metabolic pathways; these pathways are thus essential for microbial metabolism. Pyruvate metabolism, glycolysis, and amino acid metabolism dominated the carbohydrate metabolism pathways (40). Organic acids and aromatic compounds could be produced through these metabolic pathways to improve not only the fermentation quality of silage but also the palatability to animals. The silage had a high proportion of carbohydrate metabolism pathways, indicating that the LAB community dominated the silage fermentation. The bacterial relative abundance in LP1-treated silage was higher than in MO1-treated silage. Some of these microorganisms may compete with LAB for fermentation substrates and promote carbohydrate metabolism. The amino acid metabolism pathways observed in material and silage may reflect the metabolic dynamics of the dominant microbial community. Fresh materials are prone to proteolysis, which increases the proportion of amino acid metabolism pathways, as well as the content of NH_3_-N nitrogen, and reduces the flavor and quality of fermentation. In addition, through the amino acid metabolic pathway, LP1-treated silage decomposed proteins and increased the NH_3_-N content to a greater extent than MO1.

There are many species of microorganisms related to silage fermentation, and the microbial co-occurrence network system in this study was complicated. In this system, LAB and other symbiotic microorganisms promoted or restricted each other. In this study (Fig. 5), *L. plantarum* was the most dominant strain in silage and was positively correlated with *B. subtilis*. This was because *B. subtilis* is an aerobic Gram-positive spore bacteria that can survive in harsh environments, such as high temperature, acid-base, and anaerobic conditions (41). In addition, this bacteria can use the residual oxygen in the silo to proliferate in the early stage of ensiling, creating an anaerobic environment to promote the growth of lactobacilli. *Kosakonia cowanii*, *Capnocytophaga granulosa*, *Herbaspirillum* spp., and *C. pabulibutyricum* are all Gram-negative bacteria harmful to silage fermentation. Their growth consumed some of the nutrients in the silage and was negatively correlated with that of *L. plantarum*. The next most dominant bacteria was *M. trichothecenolyticum*, which relies on respiratory metabolism and produces acid from glucose and other sugars during the ensiling process. This may lower the pH and inhibit the growth of *P. agglomerans*; therefore, the growth of the two bacteria was negatively correlated.

Silage microorganisms influence the final fermentation products through a series of metabolic pathways, and these fermentation products also restrict the structure of the microbial community (42). LAB convert WSC into lactic acid through the carbohydrate metabolism pathway, lowering the pH to inhibit the proliferation of harmful microorganisms and decomposition of protein in silage, resulting in a positive correlation between *L. plantarum* and LAB or lactic acid and a negative correlation with pH or NH_3_-N (Fig. 6). Butyric acid bacteria can decompose protein through amino acid metabolism in the silage environment, increase the NH_3_-N content, and affect fermentation quality and flavor. Clostridia can decompose protein through amino acid metabolism in the silage environment. Therefore, the growth of *Clostridium butyricum* is positively correlated with NH_3_-N. Silage is the most important stored feed for ruminants. More than 20% of the total silage produced each year worldwide may be lost due to failure during preparation (43). Therefore, identifying excellent LAB strains, such as MO1, and using them as exogenous microbial inoculants can not only modulate the silage fermentation process to prepare high-quality silage but will also be important for alleviating the shortage of feed in the tropics and revitalizing the animal production industry in Africa.

## MATERIALS AND METHODS

### Silage preparation

Napier grass at the early flowering stage during the first cutting was harvested from an experimental field at the Agricultural Research Institute of Mozambique (IIAM, Matola, Mozambique) on 26 February 2019. Meanwhile, sugarcane was harvested at maturity cultivated in a field in the industrial sugar production region of Maputo, Mozambique. To prepare silage, sugarcane stems and leaves were taken approximately 1.5 m from the top, i.e., from the discarded part of the plant. The fresh materials were immediately cut into lengths of approximately 1–2 cm using a mechanical chopper (92-2S, Sida Agri-Machine Co., Ltd., Luoyang, China). The experiment used a 2 × 3 (forage × additive) factor, completely random design, and each treatment had three replicates. Silage treatments were established: control, commercial LAB inoculant (*L. plantarum* LP1, Snow Brand Seed Co., Ltd., Sapporo, Japan), and LAB strain (*L. plantarum* MO1 isolated from Mozambique). The LAB were incubated with MRS broth (Difco Laboratories, Detroit, MI, USA) in an incubator at 30°C for 24 h. The LAB culture solution was appropriately diluted with sterilized distilled water and then applied using an electronic sprayer (SSP-5H, Fujiwara Sangyo Co. Ltd., Miki, Japan). The LAB amount added was 1.0 × 10^6^ cfu/g of FM, and the control silage was sprayed with the same amount of sterilized distilled water. Approximately 150 kg of homogenized material was packed into silos, then compacted to remove air, and stored at ambient temperature (25°C–36°C). After 60 days of ensiling, samples were taken from the silos to analyze the microbial community and fermentation quality of the silage.

### Microbial analysis

The microbial population in materials and silages was determined by plate counting method (14). The LAB, aerobic bacteria, coliform bacteria, clostridia, and yeast including mold were measured by MRS agar medium (Difco Laboratories, Detroit, MI, USA), nutrient agar medium (Nissui-Seiyaku Co., Ltd., Tokyo, Japan), blue light broth agar medium (Nissui-Seiyaku Co., Ltd.), clostridia count agar medium (Nissui-Seiyaku Co., Ltd.), and potato dextrose agar medium (Nissui-Seiyaku Co., Ltd., Tokyo, Japan) at 30°C for 3–5 days. The viable microbial counts were reported as appeared microbial colonies in cfu/g of FM.

For SMRT sequencing analysis, the DNA kit (D5625-01, Omega, Norcross, GA, USA) was used for DNA extraction from the precipitate (42). The SMRTbell Express Template Prep Kit 2.0 (PacBio, Menlo Park, CA, USA) was used to prepare purified SMRTbell libraries from the amplified DNA (44). The libraries were sequenced on a single PacBio Sequel II 8M cell using the Sequel II Sequencing kit 2.0.

SMRT sequencing was performed on a PacBio RS II instrument (Pacific Biosciences, Menlo Park, CA, USA) using P6-C4 chemistry. The protocol RS_Readsofinsert.1 in SMRT Portal version 2.7 software (PacBio) was used to process the raw data (19). The SILVA database version 132 was implemented to classify different OTU and annotate the taxonomic information for each OTU representative sequence based on Bergey’s taxonomy at the genus, family, order, class, and phylum levels, according to classification at an 80% minimum bootstrap threshold. OTUs that occurred only once or twice were discarded. In order to describe the shared and unique microorganisms in all samples following the OTU clustering analyses, Venn diagrams were produced using open-source software package (version 1.2) of R statistical tools. The relative abundances of different bacterial communities at the species level were also analyzed using the Windows statistical software package. The difference between the mean values for four bacterial genera with significant changes among Napier grass silages was evaluated with ANOVA and Scheffe’s post-hoc test (α = 0.05; *n* = 3). The metabolic potential of the microbial community and the composition of functional genes were assigned to functional annotations of sequenced metagenomic sequences through 16S rRNA marker gene and were postulated based on the KEGG. The functional profiles and differences among different groups were analyzed with phylogenetic investigation of communities by reconstruction of unobserved states (PICRUSt 2). Since the Napier grass and sugarcane top silages showed similar bacterial community, we used Napier grass and silages as a representative for KEGG metabolism pathway analysis. A microbial network analysis was drawn with Python language tool. Hierarchical cluster and heat map analyses were performed using R package pvclust (version 3.0.2).

### Chemical, energy, and macro-mineral analysis

The samples before and after ensiling were dried in a 65°C oven for 48 h until a constant mass was attained. After drying, the samples were ground using a mill (T1-200, CMT Co., Ltd., Tokyo, Japan). According to the methods of the Association of Official Analytical Chemists, the DM (method 930.15), ash (method 923.03), CP (method 990.03), and EE (method 920.39) were analyzed (45). The OM content was calculated based on the weight lost after ashing. The NDF and ADF were expressed exclusive of residual ash. Heat stable amylase and sodium sulfite were used for the NDF procedure. The ADL was determined by solubilization of cellulose with sulfuric acid (46). The LBC method is to weigh 10 g of the material into 90 mL of distilled water and homogenize it with Stomacher Lab Blender (400, Seward, UK) for 5 minutes. After reducing the pH to 4.0 with HCl, the titration amount required to the pH from 4.0 to 6.0 (mmol/kg DM) with 0.1 N NaOH is used to calculate the LBC (47). The glucose, sucrose, and fructose were determined as WSC by high-performance liquid chromatography (HPLC, LC-2000 plus, JASCO Co., Tokyo, Japan) as described by Cai (28). The analytical conditions were as follows: SC 1011 column (8.0 mm × 30 cm, Shoko, Tokyo, Japan), 80°C oven, water mobile phase and 1.0 mL/min flow velocity, and Jasco RI-1530 detector.

The NPN and TP were measured by Kjeldahl analysis as per the description of Cai et al. (8). The NEm, NEl, and NEg were analyzed according to the method of Van Soest et al. (46). The macro-mineral contents of samples, including calcium, phosphorous, magnesium, and potassium, were measured using a wet-ashing method and then analyzed with an atomic absorption spectrophotometer (PerkinElmer, LAMBDA 1050, Connecticut, USA) as described by Hollis et al. (48).

### Analysis of silage fermentation

The fermentation products of the silages were analyzed using the cold water extract method (30). The 10 g of silage sample was homogenized in 90 mL of sterilized distilled water and kept in a 4°C refrigerator for 24 h. Thereafter, the extract samples were filtered through quantitative ashless filter paper (circle size: 5A, 110 mm, Advantec Co., Ltd., Tokyo, Japan). The filtrate was used to determine pH, NH_3_-N, and organic acid including lactic acid, acetic acid, propionic acid, and butyric acid. The pH was measured using a pH meter (D-71, Horiba Co., Ltd., Kyoto, Japan). The steam distillation of the filtrates was used to determine NH_3_-N using the Kjeltech auto distillation (2200, Foss Tecator, Hoganas, Sweden) as described by Cai (28). The filtrates of silages were injected into an HPLC system (LC-2000 plus, JASCO Co., Tokyo, Japan) to determine organic acid (30). The HPLC system was equipped with a Shodex RSpak column (KC-811, Showa Denko K. K., Tokyo, Japan), 60°C oven, Jasco UV-2070 detector (450 nm), 3 mM HClO_4_ eluent and reagent (0.2 mM bromothymol blue + 8 mM Na_2_HPO_4_ + 2 mM NaOH), and 1.0 mL/min flow rate.

### Statistical analysis

ANOVA was performed using the general linear model procedure of Statistical Package for the Social Sciences (SPSS version 19.0, SPSS, Inc., Chicago, IL, USA) to examine the differences between samples, and significance was declared at *P* < 0.05. The LBC, microbial population, chemical and protein composition, energy, and macro-minerals contents of samples were subjected to one-way ANOVA. Tukey’s honest significant difference test was employed for different sample means (49).

Data on the DM, fermentation quality, and microbial population after 60 days of ensiling were analyzed with a completely randomized design with a 2 × 3 (forage × additive) factorial treatment structure. The two-way ANOVA procedure of SAS version 9.1 (SAS Institute, Cary, NC, USA) was used for the analysis, and the statistical model is as follows: Y_ijk_ = μ + α_i_ + β_j_ + αβ_ij_ + ε_ijk_, where Y_ijk_ = observation; μ = overall mean, α_i_ = forage effect (i = Napier grass and sugarcane top), β_j_ = additive effect (j = 1–4), αβ_ij_ = forage × additive effect, and ε_ijk_ = error. The mean values were compared by Tukey’s test.

The network analysis showed the correlation networks among microorganisms at the species levels. The circle represents the microorganism species, the circle size represents the average abundance of the species, the line represents the correlation between the two species, the thickness of the line represents the strength of the correlation, and the color of the line: orange represents positive correlation, and green means negative correlation. The hierarchical cluster and heat map analyses showed the correlation analyses of the bacterial community with lactic acid, LAB, pH, and NH_3_-N at the species level, respectively. LAB, organic acid, pH, and NH_3_-N information are displayed horizontally, respectively, and the bacterial community information is displayed vertically. The corresponding value of the middle heat map is the Spearman correlation coefficient *r*, which ranges from −1 to 1, *r* < 0 indicates a negative correlation (blue), *r* > 0 indicates a positive correlation (red), and “*”, “**”, and “***” represent *P* < 0.05, *P* < 0.01, and *P* < 0.001, respectively.

### Conclusion

To alleviate the shortage of animal feed in the tropics, Napier grass and sugarcane top silages were prepared with screened LAB, and PacBio SMRT technology was used to analyze the microbial community, co-occurrence network, and fermentation characteristics. Sugarcane tops, like Napier grass, contain a range of nutrients. After ensiling, the main microbial community shifted from Gram-negative to Gram-positive bacteria dominated by LAB, which affected silage fermentation. The selected strain, MO1, displayed higher acid resistance and lactic acid production ability than the commercial LAB. When silage was inoculated with strain MO1, a microbial co-occurrence network system centered on *L. plantarum* was constructed, which modulated the microbial community and metabolic pathways. The selected strain MO1 can improve the fermentation quality of silage, which is of great significance for the use of local forage resources to promote the sustainable development of animal husbandry.
